# Public health communication professional development opportunities and alignment with core competencies: an environmental scan and content analysis

**DOI:** 10.24095/hpcdp.44.5.03

**Published:** 2024-05

**Authors:** Melissa MacKay, Devon McAlpine, Heather Worte, Lauren E. Grant, Andrew Papadopoulos, Jennifer E. McWhirter

**Affiliations:** 1 Department of Population Medicine, University of Guelph, Guelph, Ontario, Canada

**Keywords:** health communication, core competencies’ professional development, workforce planning

## Abstract

**Introduction:**

Communication is vital for effective and precise public health practice. The limited formal educational opportunities in health communication render professional development opportunities especially important. Competencies for public health communication describe the integrated knowledge, values, skills and behaviours required for practitioner and organizational performance. Many countries consider communication a core public health competency and use communication competencies in workforce planning and development.

**Methods:**

We conducted an environmental scan and content analysis to determine the availability of public health communication professional development opportunities in Canada and the extent to which they support communication-related core competencies. Three relevant competency frameworks were used to assess the degree to which professional development offerings supported communication competency development.

**Results:**

Overall, 45 professional development offerings were included: 16 “formalized offerings” (training opportunities such as courses, webinars, certificate programs) and 29 “materials and tools” (resources such as toolkits, guidebooks). The formalized offerings addressed 25% to 100% of the communication competencies, and the materials and tools addressed 67% to 100%. Addressing misinformation and disinformation, using current technology and communicating with diverse populations are areas in need of improved professional development.

**Conclusion:**

There is a significant gap in public health communication formalized offerings in Canada and many of the materials and tools are outdated. Public health communication professional development offerings lack coordination and do not provide comprehensive coverage across the communication competencies, limiting their utility to strengthen the public health workforce. More, and more comprehensive, professional development offerings are needed.

**Highlights**

- There have been widespread calls to transform the public health workforce in Canada.
- We conducted an environmental scan and content analysis to determine current professional development opportunities in public health communication and investigate how well they support communicationrelated core competencies.
- We found 45 professional development offerings relevant to public health communication in Canada, with varying coverage of the core competencies.
- Addressing misinformation and disinformation, the use of current technology and communicating with diverse populations are areas in need of improved professional development.
- This snapshot of the current state of public health communication professional development shows that coverage across the competencies is neither coordinated nor comprehensive.

## Introduction

With the field of public health constantly evolving due to new knowledge from research and practice and changing technology, effective communication is critical, especially during crises.^1^ Effective communication is also central to the design and implementation of public health initiatives, which impact adoption of recommended health behaviours, especially among those in underserved population groups.2 

There have been widespread calls to improve the Canadian public health system, including updating core competencies for public health and related professional development opportunities^1^,^3^-^5^ as well as public health communication.^6^,^7^ Changes in the information ecosystem have altered methods of communication and increased the threat of misinformation, undermining trust in public health communication.^8^,^9^ This is especially apparent in the context of social media, which is an important tool for delivering public health messages.^1^,^9^

Without the opportunities to continually update and adapt their communication competencies and skills, public health practitioners risk losing their credibility and the public’s trust, negatively affecting the health of Canadians.^1^ Professional development allows for the enhancement of existing skills and behaviours and acquisition of new knowledge and attitudes in order to meet workforce demands. 

In 2008, the Public Health Agency of Canada (PHAC) published *Core Competencies for Public Health: Release 1.0* (“PHAC core competencies”) after extensive consultation with public health researchers and practitioners across the country.^10^ The 36PHAC core competencies are organized into seven categories, one of which is communication.^10^ At the time of writing, the PHAC core competencies were undergoing renewal and modernization. Because of the age of the current PHAC core competencies, other public health competency frameworks may help inform Canadian public health workforce planning. 

Health Promotion Canada has a framework for discipline-specific competencies for health promotion (“HPC competencies”) based on the PHAC core competencies.^11^ The Council on Linkages Between Academia and Public Health Practice (“Council on Linkages”) in the USA has foundational core competencies for public health practitioners;^12^ these core competencies have been regularly revised since their release in 2001 and provide an up-to-date framework that reflects modern communication requirements, including addressing the infodemic and culturally appropriate communication.^12^

There are similarities across the three competency frameworks, including tailoring communication to various audiences, choosing the right communication channel(s), mobilizing communities and using technology effectively. The extent to which communication competencies from these frameworks inform professional development opportunities for public health communication is unknown.

Gaps have been identified in the public health communication courses offered by the master of public health programs in Canada.^13^,^14^ Also, research into online continuing education programs found that only about half the courses offered in 2015 included communication as a topic.^15^ Although several online courses are available for public health professionals in Canada, Jung et al.^15^ found that these did not provide comprehensive coverage of the PHAC core competencies, including within the communication domain; nor were they readily accessible through a central online database.

Given the significance of communication in public health practice and its focus in public health competency frameworks, it is important to understand the opportunities and resources currently available and how these align with the relevant competency frameworks. Identifying current professional development offerings for public health practitioners will also highlight the opportunities for building workforce competence and communication capacity. 

This current research aims to determine the availability of public health communication professional development opportunities and the extent to which they support core competencies in communication. The objectives of this research include:

- using an environmental scan to identify currently available Canadian professional development opportunities relevant to public health communication; and
- conducting a content analysis to describe how these identified professional development opportunities align with communication competencies from the relevant frameworks (PHAC core competencies, HPC competencies, and Council on Linkages competencies).

## Methods

We conducted an environmental scan to determine the current professional development landscape that supports public health communication competencies in the Canadian workforce. Our search methods were guided by previous research on competencies for public health and continuing education.^16^,^17^ Following the steps outlined by Bengtsson^18^ and Krippendorff,^19^ we analysed the content of all the professional development opportunities identified in the scan to determine their nature and the degree to which they support the development of public health communication competency.

***Search strategy***

First, the research team searched, by way of a Google site search (site:URL search terms) using the term “health communication,” the entire contents of websites of public health organizations known to them.

Next, we conducted an Internet search using the Google search engine and the following search terms: “health communication,” “public health,” ”continuing education,” “Canada.” A subsequent search used the search terms “health communication,” “public health,” “course,” “Canada.” Consistent with methodological examples and recommendations, we reviewed the first 10 pages of results of each search.^17^,^20^ The same two searches were also run using the Ontario Public Health Libraries Association custom Google search engine,^21^ the grey literature database CABI Global Health^22^ and the custom Google search engine developed by Queen’s University Library.^23^ Other resources known to the research team were also included.

***Search criteria***

Two researchers (MM and JEM) independently reviewed the professional development offerings for relevance to the following inclusion and exclusion criteria and resolved all conflicts by discussion. For a professional development opportunity to be included, it had:

- to be offered or be available within the last 12 months (materials and tools may still be available online long after their initial publication);
- to be widely available and applicable to Canadian public health practitioners;
- to reoccur as a multistep program offered to different public health organizations and/or allow repeated access through online platforms;
- to be in English;
- to be offered in Canada or be available to Canadians;
- to be relevant to Canadian public health infrastructure and governance; and
- to be related to public health communication.

Included were “formalized offerings,” that is, training opportunities such as certificate programs, courses, graduate programs, summer institutes, webinars and online learning programs, and “materials and tools,” that is, resources such as guidebooks, white papers, expert panel reports, toolkits, guidelines and briefing notes, conference proceedings, blog posts, factsheets, toolkits and websites. 

Offerings were excluded if they were single occurrence webinars, conferences or workshops; and/or limited in geographical relevance or offered in a relatively small geographical area or organization (e.g. one local public health unit). 

***Data collection***

One researcher (HW) collected the data between 13 November 2022 and 6December 2022 and recorded the information on an Excel spreadsheet.^24^ The following information was collected for each formalized offering: name, description, type (e.g. certificate program, webinar), format (e.g. hybrid, online), intended audience, time commitment, cost, the institution providing the offering, the country providing the offering and its geographical reach, date last offered, currently offered (Y/N), the URL, the search date and the search source. The following information was collected for materials and tools: title, author, description, type (e.g. guidebook, toolkit), intended audience, location, date, the URL, the search date and the search source.

***Content analysis***

The communication-related competencies from the PHAC core competencies,^10^ the HPC competencies^11^ and the Council on Linkages^12^ were used to assess the degree to which the professional development offerings support public health communication competencies (Table 1).

**Table 1:** Summary of communication competency statements from three frameworks

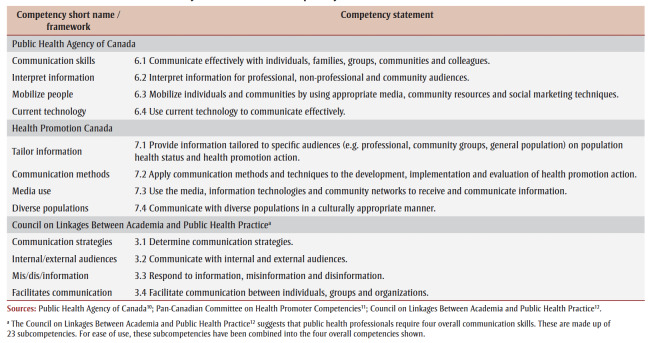

Three researchers (MM, HW and JEM) created a codebook describing key variables identified during data collection and the communication-related competencies from the frameworks described above. (This codebook is available at https://osf.io/fjtdc/.) Subvariables for each competency that reflected the named audiences, channels, tools and techniques were also captured. Professional development opportunities could be coded for the overall competency and may or may not be coded for the various subvariables depending on whether the specific audiences, channels, tools and techniques were covered. The codebook was validated prior to coding. Two researchers (HW and MM), working independently, coded the full dataset, discussing and resolving all conflicts along the way.

***Statistical analysis***

Descriptive statistics (frequencies) were calculated using Excel^24^ to assess how each of the professional development opportunities support the communication competencies. We used RAWGraphs^25^ to present the data visually.

## Results

The environmental scan uncovered a total of 45 professional development opportunities related to public health communication. Of these, 16 (36%) were formalized offerings and 29 (64%) were materials and tools. Three of the 16 formalized offerings were available and analyzed in full. The remaining 13 were analyzed based on the summary information available (most often because they were behind a paywall). All materials and tools were available and analyzed in full. 

For details on the formalized offerings and materials and tools, refer to https://osf.io/fjtdc/.

***Characteristics of professional development opportunities***

Just over half of the 16 formalized offerings (n=9; 56%) and most of the 29materials and tools (n=26; 90%) originated from Canada (Table 2). While all the formalized offerings were offered in the last 12 months (and thus met our inclusion criteria), only one (3%) set of materials and tools was published in the last 12 months and only six (21%) in the last 5 years, although all were available online within the last 12 months, thus meeting our inclusion criteria.

**Table 2:** Overview of characteristics associated with formalized offerings and materials and tools

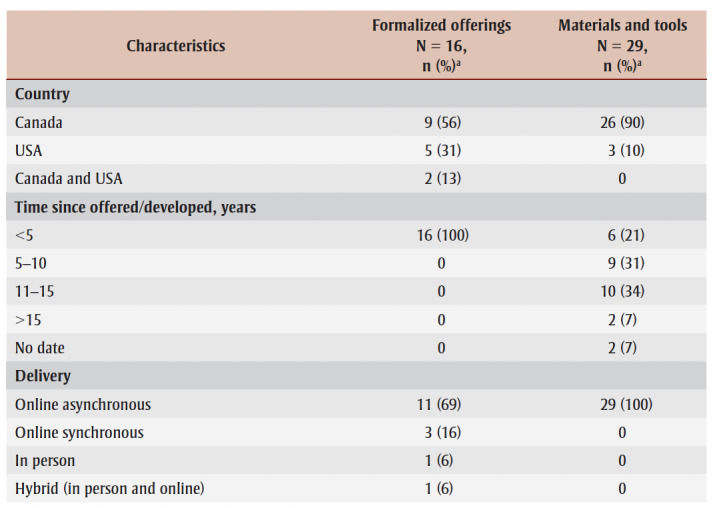 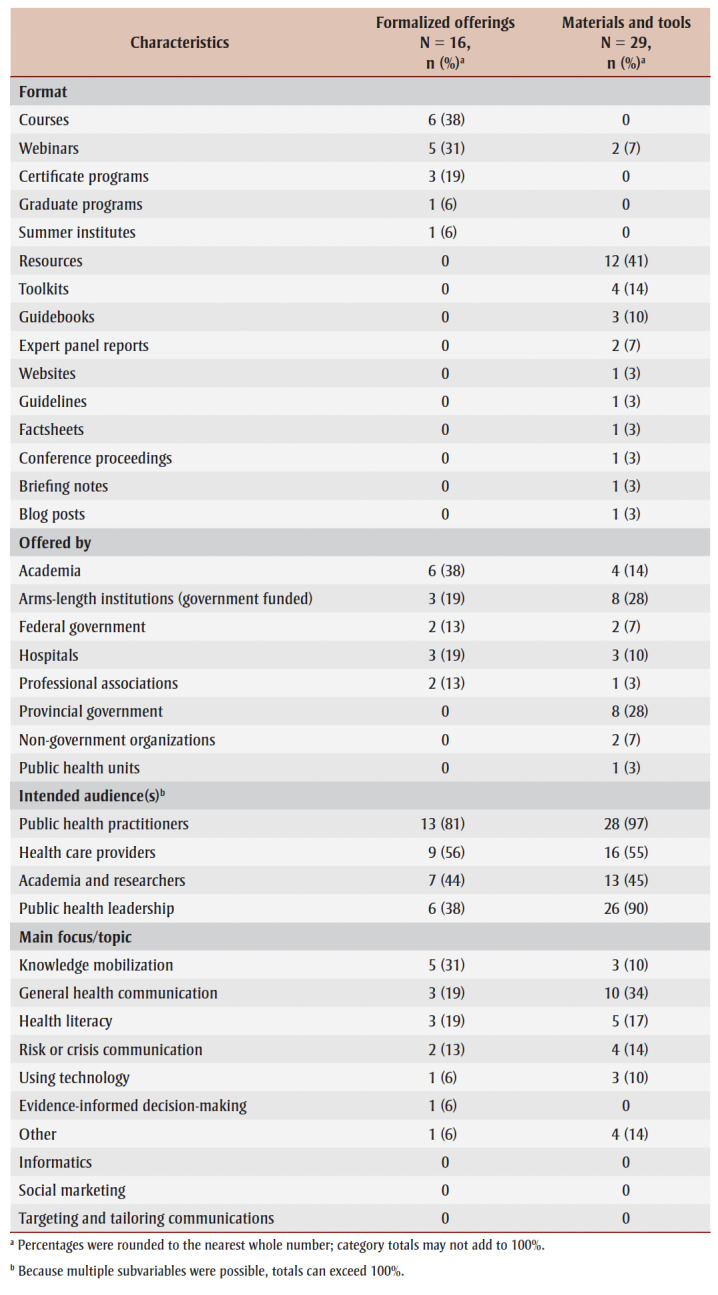

Just over two-thirds of the formalized offerings (n=11; 69%) and all the materials and tools (n=29; 100%) were offered online asynchronously; 13 of the formalized offerings (81%) and 28 of the materials and tools (97%) focused on general public health practitioners. The most common formalized offerings were courses (n=6; 38%); were focused on knowledge mobilization (n =5; 31%); and were offered by academic institutions (n=4; 14%). The most common materials and tools were resources (n=12; 41%); were focused on general health communication (n=10; 34%); and were offered by arms-length organizations (n=8; 28%).

**Figure 1:**
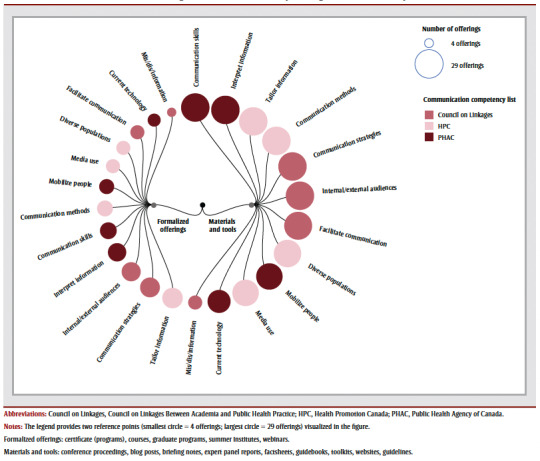
Formalized offerings and materials and tools by host organization and delivery mode

Professional development opportunities were delivered by various organizations and institutions. Formalized offerings were mostly offered by academia (n=6; 38%), government-funded arms-length institutions (n=3; 19%) and hospitals (n=3; 19%). Materials and tools were mostly offered by government-funded arms-length institutions (n=8; 28%), provincial governments (n=8; 28%) and academia (n=4; 14%) (Table 1; Figure1). No formalized offerings were provided by public health units, NGOs or provincial governments, and very few materials and tools were offered by public health units and professional associations (n=1 each; 3%) (Table 1; Figure1).

***Competencies within professional development opportunities***

**Figure 2:**
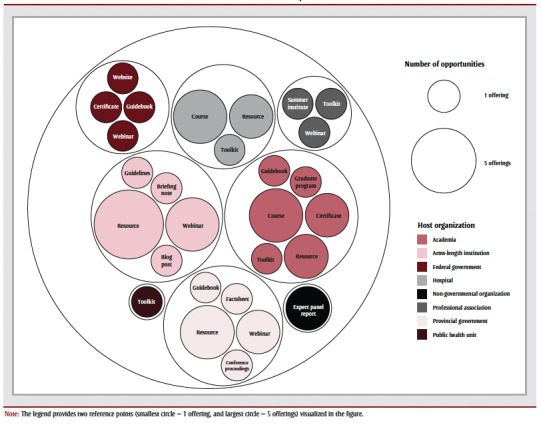
Alignment of professional development opportunities (formalized offerings and materials and tools combined) with communication competencies

Overall, across the professional development opportunities (formalized offerings and materials and tools combined; see Table 1), competencies related to tailoring information (n=44; 98%), using different communication strategies (n=43; 96%) and communicating with internal and external audiences (n=42; 93%) were the most supported; competencies related to misinformation and disinformation (n=12; 27%), current technology (n=25; 56%) and using media (n=33; 73%) were the least supported (data not shown). Figure2 shows the alignment of the professional development offerings with the competency frameworks broken down by formalized offerings (n=16) and materials and tools (n=29). 

***Alignment with PHAC communication competencies***

On average, formalized offerings covered 2.25 (range: 0–4) out of the 4 communication-related PHAC core competencies and materials and tools covered 3.55 out of these 4 competencies (range: 2–4) per professional development offering. The PHAC core competency most commonly supported by professional development opportunities was interpreting information, which was addressed by all 29 of the materials and tools and three-quarters (n=12; 75%) of formalized offerings (Table 3). Communication skills was similarly supported by all of the materials and tools and almost two-thirds (n=10; 63%) of formalized offerings. Mobilizing people was slightly less supported with 90% (n=26) of materials and tools and 50% (n=8) of formalized offerings addressing it. The least supported competency was current technology with 66% (n=19) of materials and tools and 38% (n=6) of formalized offerings addressing it.

**Table 3:** Alignment between professional development opportunities and PHAC communication competencies and subvariables including specific audiences, channels, techniques and tools in each competency

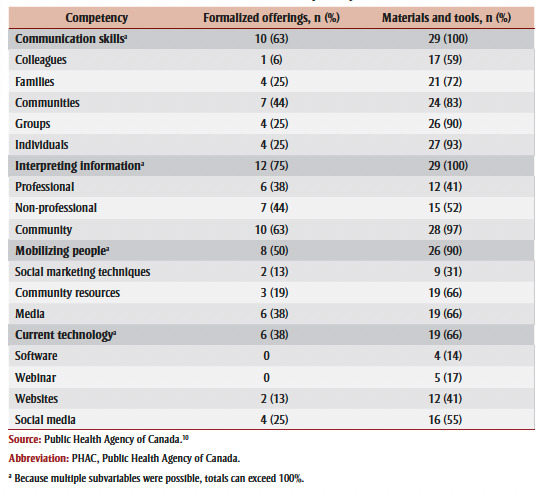

The types of intended audiences were less frequently addressed by formalized offerings compared to materials and tools (Table 3), with colleagues the least addressed audience type. Professional development opportunities most often addressed interpreting information for communities, while professional audiences were least covered. Further, social marketing techniques for mobilizing individuals and communities were not well addressed by the professional development opportunities. Finally, formalized offerings infrequently covered specific technologies identified in the competencies, while materials and tools addressed using websites and social media in approximately half of the resources that addressed this competency.

***Alignment with HPC communication competencies***

Overall, materials and tools were strongly aligned with all the HPC communication competencies (Table 4). Tailoring information to specific audiences was the most widely addressed competency by both formalized offerings and materials and tools. Coverage of the different communication methods varied, with the media (traditional and new media) addressed by seven (44%) formalized offerings and 19 (66%) materials and tools, and information technologies addressed by just two (13%) formalized offerings and 10 (34%) materials and tools. While communicating with diverse populations was well supported by formalized offerings (n=7; 44%) and materials and tools (n=27; 93%), it was often addressed exclusively in the context of health literacy (n = 2/7 [29%] formalized offerings; n=15/27 [52%] of materials and tools).

**Table 4:** Alignment between professional development opportunities and HPC communication competencies and subvariables, including specific audiences, channels, techniques and tools in each competency

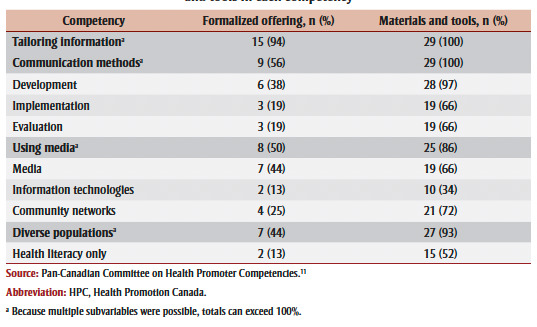

***Alignment with Council on Linkages communication competencies***

Overall, the materials and tools had more comprehensive alignment with the Council on Linkages communication competencies based on the information available, except for addressing misinformation and disinformation (Table 5). This competency subvariable had the lowest support from professional development opportunities with only four (25%) of formalized offerings and eight (28%) of materials and tools addressing misinformation and disinformation.

**Table 5:** Alignment between professional development opportunities and Council on Linkages communication competencies and subvariables, including specific audiences, channels, techniques and tools in each competency

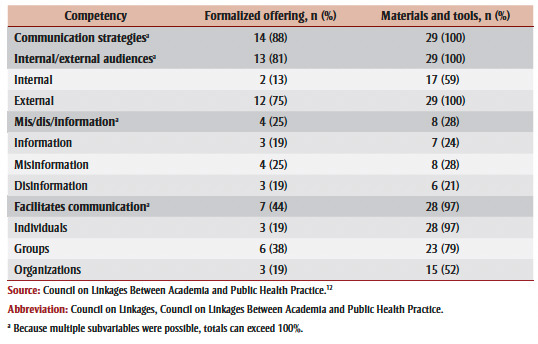

Although the remaining three competencies (communication strategies, internal/external audiences, facilitate communication) were broadly addressed by many professional development opportunities, there was less focus on some key elements. Specifically, while 17 (59%) of materials and tools addressed communicating with internal audiences, only two (13%) formalized offerings addressed this element of the competency.

## Discussion

This study examined the professional development opportunities for public health communication widely available currently or within the last 12 months, in English, to Canadians or relevant to Canadian public health, and how closely aligned they are with public health communication competencies relevant in Canada (PHAC and HPC) and the USA (Council on Linkages).

We found 45 offerings related to public health communication of which 16 were formalized offerings (training opportunities, e.g. certificate programs, courses, webinars) and 29 were materials and tools (resources, e.g. guidebooks, toolkits, reports). Less than one-quarter of the materials and tools were published in the last 5 years. The older age of some materials and tools may have contributed to the competency gaps in current technology and in addressing misinformation and disinformation. Most often, formalized offerings focussed on knowledge mobilization while materials and tools focussed on general health communication.

Professional development offerings were not developed or coordinated by a governing body, but were offered by different organizations and agencies across Canada and the USA. Overall, the formalized offerings address fewer competencies relative to the materials and tools; however, this may be, at least in part, because we were only able to analyze summary materials for the majority of formalized offerings whereas all materials and tools were available and analyzed in full.

Competencies are the integrated knowledge, skills, attitudes/values and behaviours that public health practitioners and organizations must possess for effective public health practice.^26^ Public health organizations can take competencies into account when recruiting personnel, assessing job performances and identifying professional development needs.^26^ Workforce training and continuing education are an essential part of competency development, especially when there is a lack of graduate training options in communication and other competencies, as was found in Canada.^14^,^15^ The Canadian Public Health Association has recommended workforce training in modernized competencies as key for strengthening the public health system.^1^,^27^ PHAC used to offer Skills Online, an eight-module professional development program that directly supported the core competency categories.^28^ The results of this study show that the professional development opportunities currently available do not cover all the PHAC core competencies, with formalized offerings averaging 2.25 competencies per opportunity and materials and tools averaging 3.55 competencies per opportunity. No equivalent comprehensive training program fills this gap.

Our research was specific to the public health communication categories; in fact, we found that there is no comprehensive professional development program for public health communication. What is available is a range of programs offered by many different types of organizations and agencies, some of which may not be up-to-date and which do not comprehensively support current communication competency development needs. While a comprehensive federal training program such as Skills Online^28^ may provide coordinated training across the full range of core competencies (including communication), the smaller professional development offerings could not be expected to be equally comprehensive. Rather, the professional development offerings provided by the various organizations and institutions were more targeted and not designed to cover the full range of communication competencies. Examining the professional development opportunities collectively allows for understanding what is available, how the opportunities support the development of communication competencies, and what areas of opportunity exist for public health communication in the absence of a comprehensive competency-based federal training program.

Compared to the formalized offerings, the materials and tools were more aligned with the communication-related core competencies; however, practitioners need to seek out these resources, without the benefit of a facilitated structure such as could be expected from a course. Diverse effective training includes online courses, mentorship, just-in-time training and community-engaged training, through academia, government, community and other partnerships.^29^ Materials and tools for public health communication would be less likely to reflect these pedagogical practices.

Further, recent research found that fewer than half of the master of public health programs in Canada offer courses that focus on health communication, and none specialize in health communication.^14^ As with professional development, a systematic approach to enhancing communication competence in the public health workforce is needed, and master of public health programs should include targeted health communication education taught by faculty members with the relevant expertise. In addition, curricula need to be regularly reviewed to make sure they are aligned with contemporary competencies and current public health needs.

Comprehensive professional development opportunities that address contemporary public health communication needs will strengthen our capacity and ensure the availability of a skilled workforce. In contrast to current offerings in Canada, the selection of trainings in public health communication for students and practitioners in the USA is large and comprehensive. The Public Health Foundation offers the TRAIN Learning Network; the foundation and the New England Public Health Training Center have a number of courses related to communication that are mapped to the Council on Linkages’ core competencies for public health professionals.^12^,^30^ There are also 65 schools in the USA that, between them, offer 77 programs on health communication.^31^ They could also provide a roadmap for comprehensive training and professional development aligned with core competencies and pedagogy for effective training in Canada.

Overall, the professional development offerings had strong alignment with the communication-related PHAC core competencies, with nearly half (49%) addressing all four competencies. One communication core competency, the PHAC core competency, “current technology” (#6.4), was not widely addressed by formalized offerings but had better coverage within materials and tools, although leveraging technology rather than teaching practitioners how to effectively use it tended to be mentioned. Digital technologies are vital to public health communication, as was evidenced during the COVID-19 pandemic when social media, online big data sources, data visualization, artificial intelligence and digital platforms (e.g. video conferencing software) became increasingly important.^32^ Thus, it is critical that the core competencies not only reflect the scope and complexity of digital technologies that should be used by public health in communication initiatives but also that they be mapped to professional development opportunities that teach the technologies to practitioners.

As previously mentioned, the PHAC core competencies are undergoing renewal and modernization, with an estimated launch scheduled for 2024. The Chief Public Health Officer’s 2021 report identified several areas related to communication that must be addressed through the updated competencies and workforce training: addressing misinformation and disinformation; codesigning health information with communities; culturally appropriate communication; enhanced risk and crisis communication; and tailoring information to communities’ values and needs.^33^ Our research found that most of the professional development offerings did not address misinformation and disinformation, although most did address tailoring communication and communicating with diverse populations. Within the context of communicating with diverse populations, however, the focus was usually on health literacy rather than on cultural competency. These results show opportunities for strengthening our professional development in areas vital to public health communication.

The Canadian public health workforce can be enhanced and supported by building stronger linkages between practice and education, including partnerships between public health schools and public health organizations and associations to co-develop and customize education and training opportunities, including specialized subdisciplines, for public health practitioners and students. Public health organizations and associations play key roles in workforce development and are aware of community-level needs and practitioner competencies through their connection to the field and monitoring and evaluating of key issues. As such, they are in the best position to clarify the public health needs of today and anticipate the needs of tomorrow. Public health schools, meanwhile, bring expertise in pedagogy and competency-based education. Such partnerships would help produce training opportunities that are tailored to organization and practitioner needs, including format, timing and focuses. Further, public health organizations and associations could provide comments and input to public health schools on what they anticipate needing in the future, which is important because of the lead time required to build curriculum and expertise in the future public health workforce.

***Strengths, limitations and future research***

The search strategy was designed to capture as many professional development offerings meeting our inclusion criteria as possible. The search consisted of English language results only, and some web content was inaccessible without an organization membership. Access to the full details of formalized offerings was often not possible without enrolling in the course; therefore relevant data were mostly extracted from summary information, which may have biased the results given that materials and tools often presented information in full. Further, while formalized offerings needed to be offered within the last 12months, materials and tools needed to be available online within the last year, but could have been published even 15years earlier.

Our search and data retrieval processes were also limited by challenges inherent to online research. For example, broken links due to inconsistencies in Internet archival processes were common. Also, our search results may have been biased and influenced in unknown ways by Google’s algorithms.^34^

With the PHAC core competencies currently being renewed, this environmental scan provides a valuable snapshot of what is available and how it corresponds to current communication core competencies within the health communication discipline of public health. This scan does not describe other professional development opportunities within other specializations or competency domains. Similar environmental scans of professional development offerings should be completed in the future and results assessed with the updated communication competencies.

## Conclusion

The field of public health is constantly changing as a result of new knowledge from research and practice, the changing communication ecosystem and the complexity of problems facing public health practitioners. This flux is heightening the critical role of professional development opportunities for public health practitioners to build and maintain communication-related competencies. Public health core competencies guide workforce planning, job performance assessment and professional development. These competencies are fundamental to public health capacity and contribute to improved population health. 

Our findings underscore the need for more training opportunities in public health communication and a comprehensive and coordinated approach to competency-based professional development in Canada. Although the available professional development offerings are relatively well-aligned with the PHAC core competencies, misinformation and disinformation, using current technology and communication with diverse audiences are areas with far fewer opportunities for professional development. By addressing the current gaps and aligning professional development with updated competencies, public health practitioners will be able to enhance their knowledge, values, skills and behaviours for a more effective and precise public health practice.

## Acknowledgements

Funding for this research was provided by the Canadian Institutes of Health Research (CIHR) in the form of a CIHR Catalyst Grant (FRN 184647).

## Conflicts of interest

The authors have no conflicts of interest to declare.

## Authors’ contributions and statement

MM: Conceptualization, methodology, formal analysis, funding acquisition, investigation, validation, visualization, writing– original draft, writing – review & editing. 

DM: Formal analysis, investigation, visualization, writing – original draft, writing – review & editing.

HW: Formal analysis, investigation, methodology, writing – review & editing.

LEG: Funding acquisition, writing – review & editing.

AP: Funding acquisition, writing – review & editing.

JEM: Conceptualization, formal analysis, funding acquisition, methodology, project administration, supervision, validation, writing – review & editing.

All authors have read and agreed to the published version of the manuscript.

The content and views expressed in this article are those of the authors and do not necessarily reflect those of the Government of Canada.
